# DBN-BAAE: Enhanced Lightweight Anomaly Detection Mechanism with Boosting Adversarial Autoencoder

**DOI:** 10.3390/s25103249

**Published:** 2025-05-21

**Authors:** Yanru Chen, Bei Wu, Wang Zhong, Yanru Guo, Dizhi Wu, Yi Ren, Yuanyuan Zhang

**Affiliations:** 1 College of Computer Science, Sichuan University, Chengdu 610065, China; chenyanru@scu.edu.cn (Y.C.); 2023323040022@stu.scu.edu.cn (W.Z.); guoyanru@stu.scu.edu.cn (Y.G.); 2020223045152@stu.scu.edu.cn (D.W.); 2024323040001@stu.scu.edu.cn (Y.R.); 2 Institute for Industrial Internet Research, Sichuan University, Chengdu 610065, China; 3 Pittsburgh Institute, Sichuan University, Chengdu 610207, China; 2022141520171@stu.scu.edu.cn

**Keywords:** industrial control systems, anomaly detection, deep belief network, boosting adversarial autoencoder, dynamic threshold

## Abstract

The growing digitalization of Industrial Control Systems (ICSs) presents both significant benefits and security challenges, especially for small and medium-sized factories with limited resources. Effective anomaly detection is essential to safeguard these facilities and prevent costly disruptions. Although current research has advanced anomaly detection, it is still challenging for algorithms to be capable of effectively balancing the interplay between training speed, computational cost, and accuracy while simultaneously exhibiting robust stability and adaptability. This gap often leaves small and medium-sized factories without efficient solutions. To address these issues, this work introduces a deep belief network-based boosting adversarial autoencoder termed DBN-BAAE, a novel lightweight anomaly detection mechanism based on boosting adversarial learning. The proposed lightweight mechanism saves computational overhead, enhances autoencoder training stability with an improved deep belief network (DBN) for pre-training, boosts encoder expression through ensemble learning, achieves high detection accuracy via an adversarial decoder, and employs a dynamic threshold to enhance adaptability and reduce the need for retraining. Experiments reveal that the mechanism not only achieves an F1 score of 0.82, surpassing the best baseline by 1%, but also accelerates training speed by 2.2 times, demonstrating its effectiveness and efficiency in ICS environments, particularly for small and medium-sized factories.

## 1. Introduction

In the context of Industry 4.0 [1] and initiatives like “Made in China 2025” [2], the digitalization, networking, and intellectualization of Industrial Control Systems (ICSs) [3,4] have increased significantly. This transformation brings both opportunities and challenges, especially with the growing number of interconnected devices in ICSs, which exposes systems to greater risks of security threats due to vulnerable interfaces and potential breaches [5,6]. Advanced anomaly detection mechanisms, leveraging deep learning methods, aim to improve ICS security and have made substantial progress. However, these mechanisms are primarily designed for large-scale factories [7,8]. In contrast, small and medium-sized factories face unique challenges due to limited computational resources. These factories typically have a moderate amount of computing power, including CPUs and GPUs, but they lack the high-performance infrastructure needed to train complex and computationally expensive neural networks [9]. Consequently, there is a demand for lightweight anomaly detection mechanisms that require fewer parameters, faster training times, and shorter deployment periods. Moreover, the dynamic nature of production lines and frequent changes in data patterns make it difficult for existing solutions to adapt quickly to new data features. As a result, retraining such systems can become impractical and resource-intensive. Small and medium enterprises are a noteworthy driver of economic development [10], being vital to most economies across the world, particularly in developing and emerging nations [11,12]. Thus, there is a pressing need for lightweight, efficient, and adaptive anomaly detection mechanisms that address security risks while accommodating the resource constraints of small and medium-sized factories.

Current anomaly detection algorithms for multivariate time series data can be categorized into three types: The first type comprises reconstruction-based models [8], such as autoencoder (AE) [13] and Generative Adversarial Networks (GANs) [14]. AE models are prone to overfitting, especially when dealing with noisy data, which results in reduced accuracy [15]. GANs, due to their adversarial nature, often suffer from instability, making them less reliable for consistent performance [16]. These methods, while useful, struggle to balance high computational precision with stability and efficiency, which is a critical need for real-time anomaly detection in small and medium-sized factories. The second type involves graph networks [17], which convert multivariate time series into graph structures to capture complex dependencies between dimensions and time points. Although graph networks are effective in modeling intricate relationships, they are computationally expensive due to the reliance on complex matrix operations and high-dimensional data representations. This computational overhead makes them impractical for small and medium-sized factories, where limited resources cannot afford such high computational costs. In this case, the high computational overhead compromises both efficiency and adaptability, further straining the system’s ability to operate in dynamic factory environments. The third type consists of prediction-based models [18], which apply predictive modeling to forecast time series data. Anomalies are detected by comparing real-time observations against predicted values. However, many prediction-based models use fixed models, which cannot adapt to changes in the data over time. This lack of adaptability leads to frequent retraining, which is computationally demanding and inefficient, particularly in environments with limited resources. These models struggle to balance high prediction accuracy and adaptive learning, resulting in excessive computational overhead and poor resource utilization. Simple reconstruction-based algorithms suffer from low accuracy and instability, graph networks algorithms face high computational demands, and prediction-based models lack adaptability and incur high retraining costs. In brief, existing anomaly detection algorithms struggle to balance the four key requirements: high stability, high computational precision, low computational cost, and strong adaptability. Moreover, many ICS sensors themselves operate under severe resource constraints, often limited to just a few kilobytes of SRAM and flash memory. For example, embedding a block-based Kalman-filter model in an Arduino Uno consumes 1.3 kB of its 2 kB SRAM and 13.5 kB of its 32 kB flash memory [19]. Consequently, minimizing on-sensor memory footprint and computational load must be treated as an additional, fifth design criterion for any real-time anomaly detector in industrial settings.

To address these challenges, we proposed DBN-BAAE, a new lightweight anomaly detection mechanism that combines deep belief networks (DBNs) [20] with a boosting adversarial autoencoder (BAAE). This mechanism reduces computational overhead, enhances training stability, and achieves high detection accuracy and improved adaptability with faster anomaly detection, specifically designed for small and medium-sized industrial environments with limited resources. Firstly, the instability during training is addressed by leveraging an optimized DBN for pre-training. The DBN provides the autoencoder with a good initialization, ensuring more stable training. Secondly, to address the high computational overhead of graph network algorithms, we introduced a lightweight BAAE that reduces computational overhead while maintaining effectiveness. By optimizing the architecture and reducing the complexity of the mechanism, we ensure faster anomaly detection with lower resource consumption. Thirdly, to address the challenge of detecting anomalies that closely resemble normal data and improving detection accuracy, we introduced an enhanced boosting adversarial encoder based on ensemble learning [21]. This approach enhances data feature extraction and amplifies reconstruction errors, enabling the mechanism to capture anomalies in complex ICS data more effectively, thus improving detection accuracy. Lastly, to solve the issue of model retraining due to changes in production lines in small and medium-sized factories (lack of adaptability), we proposed using dynamic thresholds to adapt to new data patterns, providing adaptability and reducing computational overhead for model retraining. Furthermore, we conducted comprehensive comparison experiments to evaluate detection accuracy and training speed. Experiments validate that the mechanism attains an F1 score of 0.82, outperforming the best baseline algorithm, and improves training by 2.2 times.

The main contributions of this work are as follows:

- DBN-BAAE framework: This work introduces DBN-BAAE, a novel lightweight anomaly detection mechanism based on boosting adversarial learning for ICS. The proposed DBN-BAAE offers not only better stability, improved training speed, but also high anomaly detection accuracy, low computational overhead, and adaptability, making it especially meet the needs of small and medium-sized factories.
- Notable performance gains: On benchmark data, DBN-BAAE achieves an F1 of 0.82, higher than all compared baseline algorithms, trains 2.2 times faster, and improves the detection time by 27% compared with the best-performing baseline algorithm.
- Fusion-driven gains: Ablation studies reveal that our fusion mechanism yields the best anomaly detection performance.

The subsequent sections are organized as follows: Section 2 discusses methods for detecting unsupervised [22] anomalies in multivariate time series [23]. Section 3 presents the motivation of our work. Section 4 elaborates on our proposed DBN-BAAE mechanism, Section 5 and Section 6 present the experimental details and showcase the state-of-the-art performance of our mechanism, respectively.

## 2. Related Work

This section reviews anomaly detection methods for multivariate time series, categorizing them into reconstruction-based, graph network-based, and prediction-based approaches. Reconstruction-based approaches detect anomalies by measuring reconstruction error. Graph network-based methods build relational graphs over variables. Prediction-based models forecast future values and flag deviations. To better reflect the strengths and weaknesses of each category, we explicitly compare them in terms of detection accuracy, training stability, computational cost, and adaptability: reconstruction-based models provide interpretability but often overfit noisy data; graph network-based methods capture inter-series dependencies with high precision yet incur heavy memory use and slow convergence; prediction-based models train quickly and support online forecasting but struggle with long-range dependencies and adapting to shifting data patterns.

Li et al. [24] introduced a hidden Markov model that transforms multivariate anomaly detection into univariate anomaly detection. However, this method results in ineffective detection of anomalies. Tuli et al. [25] proposed a deep transformer-based model TranAD using Model-Agnostic Meta-Learning (MAML) for fast training and large sequence processing, but the detection accuracy needs improvement. Lee et al. [26] used a lightweight Long Short-Term Memory (LSTM) [27] model based on historical data points to predict abnormal data, but the detection accuracy needed improvement. These methods have scope for improvement in detection accuracy and exhibit training instability, which limits their effectiveness in reliably identifying anomalies.

Su et al. [28] introduced a stochastic recurrent neural network model OmniAnomaly which combines Gated Recurrent Unit (GRU) [29] and Variational Autoencoder (VAE) [30], using the reconstruction probability to make anomaly judgments, but the computational complexity of the algorithm is high. Li et al. [31] devised a GAN-based method using a dilated convolutional transformer (DCT-GAN), though it suffers from a time-consuming process. Yu et al. [32] designed the maximum information coefficient attention graph network (MAG), which combines a graph neural network, embedding vectors, and LSTM, but computational efficiency may need optimization. Zhao et al. [33] used graph attention networks to extract connections in multivariate time series, but the convergence time is relatively high. These models, although effective, have high computational costs, which limit their feasibility for anomaly detection tasks in resource-constrained environments.

Park et al. [34] combined LSTM with a VAE for anomaly detection, using reconstruction error and probability but with limited adaptability. Munir et al. [35] proposed DeepAnT, a fixed Convolutional Neural Network (CNN) [36] that predicts future values, but this model lacks adaptability to changing patterns in the ICS data. Park et al. [37] proposed a model combining CNN with autoencoders. Pietroń et al. [38] optimized automatic encoders using genetic operators to reduce training data and improve accuracy without sacrificing speed, yet the model’s adaptability could be enhanced to handle dynamic data patterns better. These methods struggle to adapt to changing data patterns, limiting their applicability in environments with evolving anomaly characteristics.

Despite these advances, no existing method simultaneously delivers high accuracy, robust stability, low computational overhead, and adaptability, particularly under the resource constraints of small and medium-sized factories. This gap motivates our proposed DBN-BAAE model, which combines boosting adversarial learning with a compact architecture to achieve fast convergence, low resource use, and reliable anomaly detection across evolving data patterns. See Table 1.

## 3. Motivation

The increasing digitalization of ICSs brings both significant benefits and security challenges, particularly for small and medium-sized factories with limited resources, where effective anomaly detection is crucial to ensure security and prevent costly disruptions. While existing anomaly detection solutions are often tailored for large factories, they are impractical for small and medium-sized factories due to high computational demands and complexity. These factories face challenges such as limited computing power, making it difficult to deploy complex models, and frequent changes in production lines, which require adaptable systems. Current solutions struggle to achieve the critical balance between high stability, adaptability, low computational overhead, and detection accuracy. Therefore, there is a pressing need for lightweight, adaptive anomaly detection mechanisms that provide accurate, efficient security while accommodating the resource constraints and changing environments of small and medium-sized factories.

## 4. Method

### 4.1. Overview

**Figure 1:**
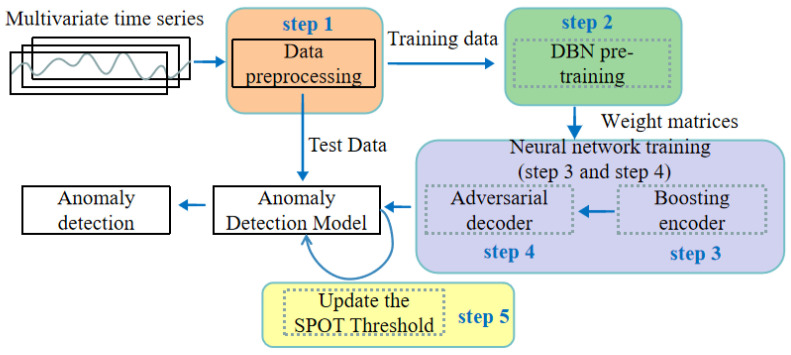
Algorithm overview schematic.

The proposed deep belief network-based boosting adversarial autoencoder, termed DBN-BAAE, is a novel lightweight anomaly detection mechanism for ICSs based on a boosting adversarial autoencoder, as shown in Figure 1. It consists of five main steps, with the dashed line highlighting the innovations of this work. Unlike typical anomaly detection methods, this work introduces a DBN pre-training step before neural network training to enhance algorithm stability. Additionally, a two-stage training process with a boosting encoder and adversarial decoder is proposed, simplifying the structure and improving detection accuracy. Finally, during the anomaly detection phase, the Streaming Peaks Over Threshold (SPOT) [39] threshold is dynamically updated based on the test data, enhancing adaptability.

The first step, data pre-processing, involves normalizing time series data from factory sensors and applying the sliding window technique [40] to create time series windows for easier training and detection.

The second step, enhanced DBN pre-training, involves maximizing stability by pre-training with an enhanced DBN model to provide a good initial state for the autoencoder. This enables the autoencoder to find a better minimum during training, enhancing training stability.

The third step, enhanced boosting encoder training, is required in order to improve the reconstruction ability of the autoencoder while maintaining its simplicity. The enhanced boosting encoder utilizes the weight matrices learned during the DBN pre-training as a starting point to construct *m* deep autoencoders and two decoders. These components are trained iteratively using ensemble learning techniques, allowing the model to learn multiple representations of the data, thereby improving its overall performance and robustness.

The fourth step, adversarial decoder training, involves amplifying data reconstruction errors using an adversarial approach with Decoder1 as the generator and Decoder2 as the discriminator.

The fifth step, dynamic threshold for anomaly detection, improves the model’s adaptability. The dynamic threshold is proposed based on test data anomalies, which are updated according to the results.

### 4.2. Enhanced DBN Pre-Training

In this subsection, we discuss the second step of the proposed DBN-BAAE mechanism, enhanced DBN pre-training. To improve feature extraction and stabilize convergence of autoencoders, we propose using an enhanced DBN for pre-training. A DBN is a multi-layer neural network model that learns feature representations layer-by-layer using a restricted Boltzmann machine (RBM) [41] with multiple hidden layers. The pre-training process initializes network parameters layer-by-layer through greedy unsupervised training [42], making optimization easier. This process, using an RBM, employs the Contrastive Divergence (CD) algorithm [43].

In the first step, initializing network parameters, we set weights and biases for each layer. In DBN, the first layer is the visual layer, the last is the output layer, and the intermediate layers are hidden layers.

In the second step, training the first RBM, we feed data into the first layer’s RBM, perform several CD iterations, and update the weights and biases.

In the third step, we train subsequent RBMs. Using the output of the previous layer as input for the current layer, we perform multiple CD iterations, and update weights and biases.

By using DBN pre-training, the autoencoder is effectively guided toward a better local minimum in the parameter space. This is because each RBM layer refines the feature representations, reducing the likelihood of getting stuck in poor local minima when the autoencoder is subsequently fine-tuned.

#### 4.2.1. Improved Parameter Initialization Method

To achieve rapid convergence and ensure training stability in a DBN, we propose a combined approach of random initialization and greedy layer-by-layer initialization. Proper parameter initialization, including weight matrices, bias vectors, learning rate, number of network layers, and nodes, is essential for improving convergence time and stability.

Random initialization provides each layer’s weights and biases with random values before training. While random initialization is quick, it can lead to fluctuations during early training steps, thereby slowing down convergence. To mitigate the drawbacks of purely random initialization, we apply greedy layer-by-layer training using RBMs. Specifically, we train each RBM on top of the output (hidden representation) of the previous RBM. This approach stabilizes the parameter space by progressively learning meaningful features, ensuring that each subsequent layer starts from a better-informed initialization rather than purely random values.

#### 4.2.2. Improved Adaptive Learning Rate Approach

To efficiently approximate the probability distribution of real data in RBM training, we use the Contrastive Divergence-*K* (CD-*K*) algorithm instead of stochastic gradient descent alone. CD-*K*, a Markov Chain Monte Carlo (MCMC) [44] sampling algorithm, starts by selecting an initial state and then simulates the Markov process through cyclic sampling. This approach helps achieve a smooth distribution, overcoming the limitations of requiring numerous Gibbs sampling cycles.

Traditional CD-*K* uses a fixed learning rate, which remains constant regardless of parameter update direction. In contrast, adaptive learning rates adjust dynamically based on update direction, helping RBMs avoid local minima and improving performance. However, previous global adaptive learning methods applied a uniform learning rate across all DBN levels, which may not optimize all RBMs effectively.

##### Fine-Grained Adaptive Learning Rate

We propose an enhanced fine-grained adaptive learning rate to accelerate RBM training:

(1) $$\eta =\left\{\begin{array}{cc} \alpha \eta_{0}, & \Delta \theta \cdot {\left(\Delta \theta \right)}^{\prime }>0,\\ \beta \eta_{0}, & \Delta \theta \cdot {\left(\Delta \theta \right)}^{\prime }<0, \end{array}\right.$$

where α and β are two hyperparameters (α>1 is the increment factor and β<1 is the decrement factor). Here, $\eta_{0}$ denotes the initial learning rate, which controls the magnitude of weight updates before adaptation and Δθ and ${\left(\Delta \theta \right)}^{\prime }$ are consecutive weight updates. These updates are computed as

(2) $$\Delta \theta =E_{\mathrm{data}}{\left(v_{i}h_{j}\right)}_{0}-E_{\mathrm{model}}{\left(v_{i}h_{j}\right)}_{k},$$

(3) $${\left(\Delta \theta \right)}^{\prime }=E_{\mathrm{data}}{\left(v_{i}h_{j}\right)}_{0}^{\prime }-E_{\mathrm{model}}{\left(v_{i}h_{j}\right)}_{k},$$

where *k* is the number of Gibbs samples and E(·) denotes the average output of Gibbs sampling. In practice, $E_{\mathrm{data}}\left(v_{i}h_{j}\right)$ is obtained by computing the expectation of the product $v_{i}h_{j}$ under the empirical data distribution, whereas $E_{\mathrm{model}}\left(v_{i}h_{j}\right)$ is the corresponding expectation under the model distribution after *k* steps of Gibbs sampling. At each training iteration, we compute Δθ (for the current update) and ${\left(\Delta \theta \right)}^{\prime }$ (for the previous update) for each layer. The DBN then checks the sign of the dot product $\Delta \theta \cdot {\left(\Delta \theta \right)}^{\prime }$ to decide whether to increase or decrease the learning rate. If the signs of these updates are the same, the learning rate increases to accelerate convergence; otherwise, it decreases to avoid overshooting. This adaptive mechanism is applied individually to each layer, allowing fine-grained control over each RBM’s training.

#### 4.2.3. Evaluation and Stability Analysis

Enhanced DBN pre-training provides the autoencoder with more discriminative initial features. By training RBMs layer-by-layer, the DBN builds a hierarchy of features that guides the autoencoder toward a better local minimum, resulting in more stable and reliable convergence compared to training without DBN pre-training.

**Figure 2:**
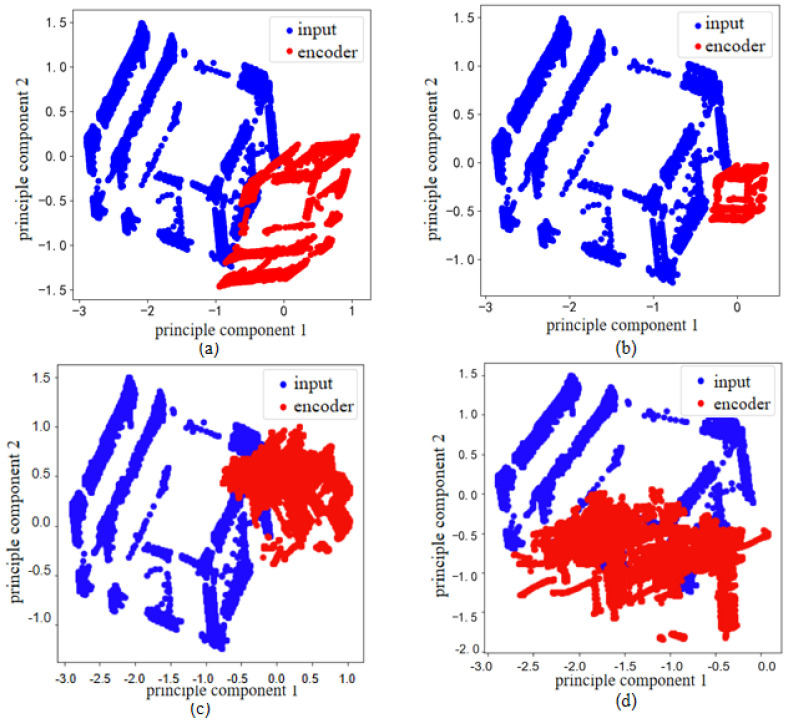
Two dimensional expansion of features. (**a**) Initial training stage without DBN pretraining. (**b**) Final training stage without DBN pretraining. (**c**) Initial training stage with DBN pretraining. (**d**) Final training stage with DBN pretraining.

To highlight the importance of enhanced DBN pre-training, we compare autoencoders with and without DBN pre-training. Using the dataset from the last day, we visualize the data after applying Principal Component Analysis (PCA) [45] to extract two principal components during autoencoder training, shown in 2D plots in Figure 2. The blue points represent the input data projections, and the red points represent the feature projections learned by the autoencoder.

- Without DBN pre-training: Figure 2a,b show the feature projections at the start and end of training. Although there is some clustering in the red points, the spread is still relatively large in Figure 2b, indicating higher variance and less stable convergence.
- With DBN pre-training: Figure 2c,d show that even at the start of training (Figure 2c), the feature projection (red points) is already more compact and better structured. By the end of training (Figure 2d), the red points converge to a much denser and more stable cluster.

Comparing Figure 2c and Figure 2d demonstrates the stability of our approach: the feature distribution in Figure 2d remains tight and less scattered, indicating that the model parameters have settled into a stable region with lower reconstruction error. In contrast, without DBN pre-training, the autoencoder’s parameters often oscillate longer before converging, as seen in the relatively dispersed feature distribution of Figure 2b.

**Figure 3:**
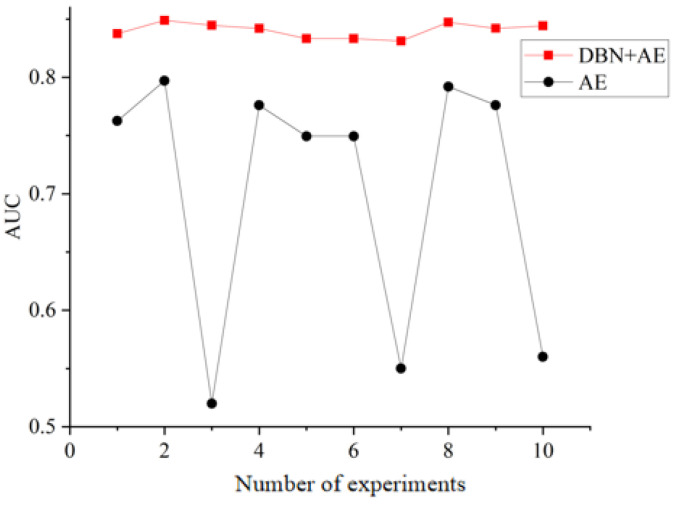
Comparison diagram of training control group.

To assess the impact of the extracted features on anomaly detection, we compare the AUC scores of autoencoders with and without DBN pre-training, as shown in Figure 3. In Figure 3, the AE-only model experiences sharp AUC declines to around 0.50 in experiments 3, 7 and 10. These performance dips arise from the stochastic nature of weight initialization and from certain noise realizations that can trap a single autoencoder in a suboptimal reconstruction result. Consequently, its ability to distinguish normal from anomalous patterns is affected. The pre-trained model exhibits more stable and consistent performance across multiple experiments, demonstrating that enhanced DBN pre-training improves the overall stability of autoencoder training and its effectiveness for downstream anomaly detection.

### 4.3. Neural Network Training

In this subsection, we discuss the third step of the proposed DBN-BAAE mechanism, enhanced boosting encoder training, and the fourth step, adversarial decoder training.

**Figure 4:**
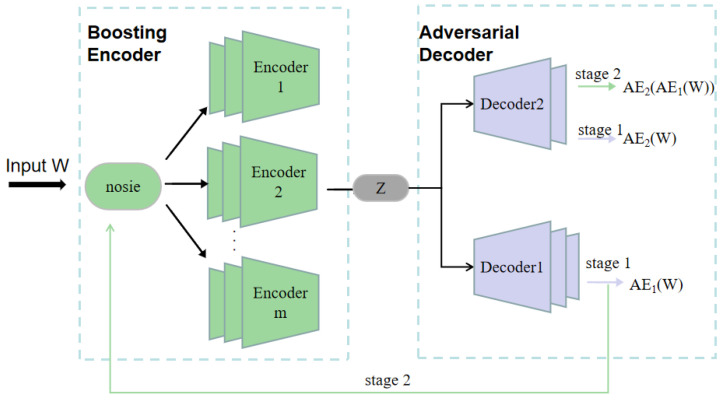
Neural network structure.

Neural network [46] training involves two steps, as shown in Figure 4. First, an enhanced boosting encoder is used. Since simple encoders have limited capability with multidimensional data, multiple integrated encoders are employed to improve feature extraction. This approach also mitigates issues like false outliers and feature loss caused by the mean function in the output. Second, an adversarial decoder structure is introduced. Traditional encoder–decoder models struggle with detecting anomalies close to normal values, reducing accuracy. To address this, adversarial learning is incorporated at the decoder stage, enhancing its ability to distinguish real data and improving detection accuracy. In Figure 4, the horizontal axis denotes the sequential flow of data: raw input *W* to boosted encoder branches to latent code *Z* to two-stage adversarial decoding (AE_1_ then AE_2_).

#### 4.3.1. Enhanced Boosting Encoder Training

Ensemble learning uses multiple decision models instead of a single one to reduce bias and variance in the classifier [21]. In this algorithm, the encoder layer comprises *m* encoders, each maintaining a distribution function of data points to guide the focus of the next encoder. The weight of the distribution function is updated based on the mean square error between the input and the decoder output, as shown in Equation (4):

(4) $$P_{x}^{i}=\alpha e_{x}^{i-1},$$

the reconstruction error of the data point *x* in the (i−1)th encoder is denoted as $e_{x}^{i-1}$, with a positive hyperparameter α to prevent excessively large weights. Data points with higher reconstruction errors are more likely to be sampled in the next training, allowing further learning. This approach combines multiple autoencoder results, focusing on hard-to-reconstruct data and reducing the risk of overfitting to normal data.

However, during early computation, some data points may have large initial reconstruction errors, which are normal at the initialization stage and not true anomalies. Simple sampling based on reconstruction error can cause the model to repeatedly select these points, leading to overfitting and high false alarm rates on new data. Averaging outputs of multiple autoencoders can also lead to ignoring important contributions from some, reducing detection performance. To address these issues, we introduce an enhanced boosting encoder. To mitigate early false anomalies, we employ weighted sampling that equally focuses on both abnormal and normal samples, thereby enhancing feature learning. Additionally, we refine the weight update formula by incorporating two distinct probability measures to emphasize both normal and abnormal samples. The Normal Probability (NP) is designed to prioritize normal samples and is defined in Equation (5) as

(5) $$NP_{x}^{i}=\frac{1/e_{x}^{i-1}}{\sum_{j=1}^{i}1/e_{x}^{j-1}},$$

where $e_{x}^{i-1}$ is the reconstruction error of sample *x* obtained from the (i−1)th encoder. This formulation is inversely proportional to the reconstruction error, thereby assigning a higher probability to samples that are easier to reconstruct.

In contrast, the Anomaly Probability (AP), introduced in Equation (6), aims to emphasize abnormal samples by being directly proportional to the reconstruction error:

(6) $$AP_{x}^{i}=e_{x}^{i-1}\sum_{j=1}^{i}1/e_{x}^{j-1}.$$

Here, a higher reconstruction error leads to a higher probability, highlighting samples that are more difficult to reconstruct and are more likely to be anomalies.

It is important to note that while both $NP_{x}^{i}$ and $AP_{x}^{i}$ use a common normalization term (i.e., the sum of the reciprocals of the reconstruction errors), they serve complementary roles in the training process. The Normal Probability (NP) focuses on retaining the information of normal samples, whereas the Anomaly Probability (AP) increases the model’s attention to potential anomalies. This dual approach helps balance the learning process, preventing overfitting to either normal or abnormal data. By combining these, the update probability (UP) for each data point is obtained as

(7) $$UP_{x}^{i}=\lambda^{-i}NP_{x}^{i}+\left(1-\lambda^{-i}\right)AP_{x}^{i},$$

$UP_{x}^{i}$ represents the probability of selecting data point *x* during the (i)th training iteration. A training parameter λ, close to one, initially emphasizes NP over AP, focusing on normal samples to prevent underfitting when false outliers are prevalent. As training progresses and false outliers decrease, NP decreases while AP increases, enabling the model to better fit real outliers. This strategy prevents early underfitting and ensures the model learns challenging samples later, improving robustness and accuracy.

Moreover, instead of a simple average which treats all encoders equally and may dilute specialized features learned by high-performing encoders, we employ a weighted fusion strategy. We define the weight

(8) $$W_{i}\left(x\right)=\frac{\frac{1}{\sum_{j=1}^{i}e_{x}^{j}}}{\sum_{k=1}^{m}\left(\frac{1}{\sum_{j=1}^{k}e_{x}^{j}}\right)}.$$

The fused feature representation is then computed as a convex combination of individual encoder outputs $z_{i}$:

(9) $$z=\sum_{i=1}^{m}W_{i}\left(x\right)\;z_{i}.$$

Theoretical justification for this weighted fusion arises from ensemble learning theory [47], which shows that convex combinations weighted by individual model reliability minimize ensemble variance and bias. By assigning higher weights to encoders with smaller reconstruction errors, the fused representation emphasizes more reliable feature mappings, yielding a richer and more discriminative feature space than uniform averaging or a single autoencoder.

To further enhance feature robustness during detection, a Gaussian noise layer is added before training, as represented by Equation (10):

(10) $$x^{\prime }=x+\varepsilon ,\;\varepsilon ∼N\left(0,\sigma^{2}\right),$$

where $\sigma^{2}$ controls the noise variance. The theoretical benefits of Gaussian noise injection are well documented:

Decision-boundary smoothing. From the Vicinal Risk Minimization perspective [48], sampling in a local neighborhood around each training example enforces Lipschitz continuity of the learned function, improving generalization to unseen perturbations.

Robust feature learning. In autoencoder-based models, noise injection yields a denoising effect—akin to contractive autoencoders—that promotes stable representations under input variations [49].

The proposed method involves adding a Gaussian noise layer to the original data *x*, where ε is the noise vector obtained through Gaussian sampling, and $x^{\prime }$ represents the noise-processed data. The pseudo-code for this procedure is presented in Algorithm 1.    
**Algorithm 1:** Enhanced boosting encoder training
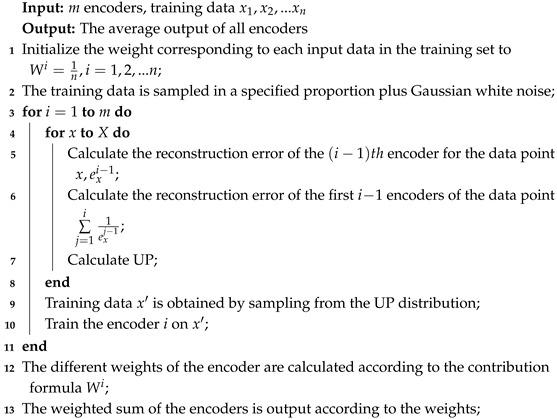


#### 4.3.2. Adversarial Decoder Training

**Figure 5:**
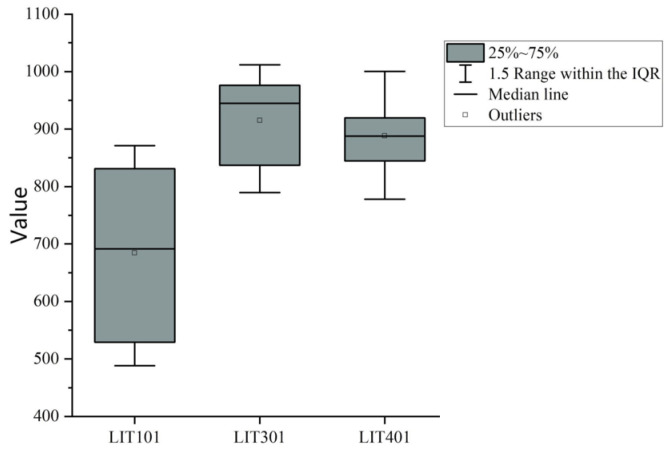
Key sensor box diagram.

To highlight the importance of adversarial training, an analysis of data characteristics is conducted. Three representative data dimensions, LIT101, LIT301, and LIT401, are selected from key sensors. Abnormal data from the past four days, including attack points, are depicted in a box diagram (Figure 5), revealing that these anomalies cluster closely around the median, making them hard to detect. To address this, an adversarial decoder is proposed. This decoder allows $AE_{2}$ to determine if data generated by $AE_{1}$ is real during adversarial learning, improving the identification of true anomalies and amplifying reconstruction errors. Inspired by the adversarial training introduced by Goodfellow et al. [14] with GANs, this method enhances model robustness. GANs, consisting of a generator and a discriminator, are trained against each other: the generator creates fake data, while the discriminator identifies it. Instead of directly using GANs, we apply adversarial training to the existing decoder, allowing for detecting anomalies close to real data while keeping the architecture simple.

A deep $AE_{1}$ and a shallow $AE_{2}$ are defined, with $AE_{1}$ acting as a strong generator that creates realistic data to deceive $AE_{2}$. This enables $AE_{2}$ to detect anomalies that closely resemble normal data, thereby enhancing detection accuracy. During training, $AE_{1}$ generates fake data to confuse $AE_{2}$ by minimizing the difference between $AE_{2}$ and *W*. Meanwhile, $AE_{2}$ is trained to distinguish real data from $AE_{1}$’s reconstructions, maximizing the difference with *W*. The formulas for each decoder are given in Equations (11) and (12):

(11) $$\varphi AE_{1}=+||W-AE_{2}\left(AE_{1}\left(W\right)\right)|{|}_{2},$$

(12) $$\varphi AE_{2}=-||W-AE_{2}\left(AE_{1}\left(W\right)\right)|{|}_{2},$$

the overall loss function loss is shown in Equation (13):

(13) $$loss=min_{AE1}max_{AE2}||W-AE_{2}\left(AE_{1}\left(W\right)\right)|{|}_{2}.$$

#### 4.3.3. Merging Training

To streamline training and reduce iterations, we propose merging the training of the enhanced boosting encoder and adversarial training into a single stage. In the original two-stage process, the decoder is used in both stages. Specifically, $AE_{1}$ first minimizes the reconstruction error of input *W* and then minimizes the difference between its reconstruction error and $AE_{2}$’s. Similarly, $AE_{2}$ first minimizes the reconstruction error of input *W*, then maximizes the difference between its reconstructed data and $AE_{1}$’s true data. To simplify and conserve computational resources, we combine these stages into a single iteration with the merged objective functions defined in Equations (14) and (15).

(14) $$\varphi AE_{1}=\lambda^{-n}||W-AE_{1}\left(W\right)|{|}_{2}+\left(1-\lambda^{-n}\right)||W-AE_{2}\left(AE_{1}\left(W\right)\right)|{|}_{2},$$

(15) $$\varphi AE_{2}=\lambda^{-n}||W-AE_{2}\left(W\right)|{|}_{2}-\left(1-\lambda^{-n}\right)||W-AE_{2}\left(AE_{1}\left(W\right)\right)|{|}_{2}.$$

During training, a parameter λ, close to one, is set. Initially, when n=1, the adversarial training weight is nearly zero, giving priority to encoder reconstruction to ensure stability. High early reconstruction loss makes adversarial data inaccurate, so the adversarial loss weight is kept low. As the autoencoder stabilizes and the reconstruction data aligns with the input, adversarial data accuracy improves. Increasing *n* raises the adversarial loss weight accordingly. An exponential function $\lambda^{-n}$ with a small positive parameter is used for weighting, similar to adaptive parameters in the boosting encoder, optimizing performance across training stages. Intuitively, during the very first iterations, $\lambda^{-n}$ remains close to 1 so the reconstruction loss term dominates (adversarial weight <0.1), which stabilizes gradient updates and allows the autoencoder to learn a smooth manifold of normal data. As training continues, $\lambda^{-n}$ decays to around 0.5, balancing reconstruction and adversarial pressures, which sharpens latent representations around emerging anomaly boundaries. In later iterations, $\lambda^{-n}$ falls below 0.3, so the adversarial loss term dominates (weight > 0.7), driving the model to discriminate subtle anomalies and produce more discriminative features. This gradual shift prevents early training oscillations while ensuring stronger feature extraction in later epochs.

### 4.4. Dynamic Threshold

In this subsection, we discuss the fifth step of the proposed DBN-BAAE mechanism, the dynamic threshold for anomaly detection.

The multivariate time series deep learning anomaly detection framework includes offline learning and online detection. Offline training produces the anomaly detection model, which is then used for real-time detection. In the online phase, the model calculates anomaly scores for new data and compares them to a threshold. If the score exceeds the threshold, the data is flagged as anomalous. A concise anomaly score formula ensures flexibility across scenarios, and setting the right threshold is crucial: too low triggers false alarms, while too high leads to missed detections. Given the changing data patterns in industrial settings, frequent retraining is impractical, so a dynamically adjustable online threshold is proposed.

To improve model adaptability to changing data and reduce retraining costs, a dynamic threshold method using Extreme Value Theory (EVT) [39] is introduced. EVT allows inference of extreme events via probability functions without assuming a specific data distribution. According to EVT’s Peak-over-Threshold (POT) theory, exceedances beyond a threshold follow the Generalized Pareto Distribution (GPD). The GPD formula is provided in Equation (16).

(16) $$F_{GPD}\left(x;\mu ,\sigma ,\xi \right)=1-{\left(1+\frac{\sigma }{\xi }\left(\frac{x-\mu }{\sigma }\right)\right)}^{-1/\xi },$$

POT defines the extreme value $Z_{q}$ as the probability of a sample exceeding a small value *q*, with a constant *t* set at the 98th percentile of the data. Peaks above *t* but below $Z_{q}$ are considered normal. GPD parameters are estimated using maximum likelihood to determine the threshold $Z_{q}$ for anomaly detection. For real-time detection, the POT algorithm is adapted into the SPOT method, which follows these steps:

1. Initialization: Use POT on the first *n* values to set the initial threshold $Z_{q}$.
2. Streaming Detection/Update: For each new score $s_{t}$, if $s_{t}>Z_{q}$, mark it as an anomaly and add to set *S*. If $t<s_{t}\leq Z_{q}$, treat it as normal and update the threshold by re-estimating GPD parameters on the most recent *m* non-anomalous scores. If $s_{t}\leq t$, discard from the update to avoid bias from normal baseline noise.

To integrate this threshold into the online anomaly detection stage, we apply a sliding window of size *m* to the incoming scores and re-fit the GPD only when scores fall between *t* and the current $Z_{q}$. This ensures that the threshold adapts smoothly to both abrupt spikes and gradual drifts in the underlying data stream.

Parameter Sensitivity and Validity: We provide the following intuitive guidance on parameter selection, which holds across a variety of industrial time series:

Percentile *q*: Choosing *q* closer to 100% makes the threshold more conservative—better at avoiding false positives but possibly slower at catching subtle anomalies. A slightly lower *q* increases sensitivity to smaller deviations.

Window size *m*: A larger window smooths out short-term fluctuations but may delay adaptation when the process shifts rapidly. A smaller window enables quicker response at the risk of over-reacting to noise.

Baseline cutoff *t*: Setting *t* at the 95th–98th percentile balances the exclusion of routine fluctuations against the inclusion of meaningful extreme values for the update.

Although the model targets complex multidimensional industrial data, it converts this data into univariate anomaly scores through reconstruction and anomaly calculation.

**Figure 6:**
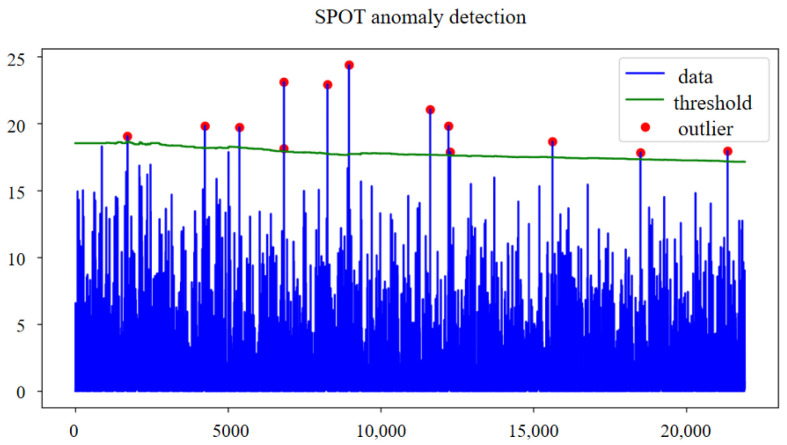
SPOT dynamic threshold variation diagram.

To demonstrate SPOT’s effectiveness, we selected a period of LIT501 sensor data during an attack, characterized by significant outlier fluctuations. Figure 6 shows the dynamic thresholds and original data using the SPOT algorithm. The SPOT thresholds continuously update with the data stream, effectively detecting anomalies and reducing false positives, making it ideal for real-time factory scenarios and adapting to the changing data in small and medium-sized factory environments.

## 5. Experiments

This section first details the experimental setup, including hardware and software configurations, dataset description, anomaly detection metrics, and baseline algorithm introductions. It then covers experimental analysis, including results, comparisons, and ablation experiments.

### 5.1. Experimental Setup

#### 5.1.1. Implementation Details

The software environment for this work includes tensorflow-gpu version 1.12.0, CUDA 10.0, Python 3.8, and ubuntu16.04 OS. The hardware configuration consists of an RTX 2080 GPU (11 GB), an Intel(R) Xeon(R) Platinum 8255C CPU @ 2.50 GHz, and 43 GB of memory. Also, hyperparameter values are provided.

In our experiments, we used a sliding window of length 12 and set the latent-space dimension to 100. The RBM network was trained with a learning rate of 0.001, performing 5 rounds of Gibbs sampling per update (K = 5). The encoder comprises layers with dimensions of 500 to 250 to 100, and the decoder mirrors this structure in reverse: 100 to 250 to 500. To balance normal and abnormal samples during training, we employed a dynamic weighting factor of λ=0.95. Finally, each training iteration processed a batch of 7919 samples.

#### 5.1.2. Datasets

The dataset used is Swat [50], a small water treatment test-bed that produces 5 gallons of filtered water per minute. It closely resembles a scaled-down version of a modern large-scale water treatment system. Developed with PUB Singapore, it simulates actual field systems, making the results applicable to real-world scenarios. Swat is widely used to study network attacks and system responses, aiding in designing new ICS security countermeasures. The dataset, in CSV format, contains 495,001 data lines, each with a timestamp and 51 sensor measurements at specific time points. The SWaT dataset is recorded at 1-s intervals, so each consecutive timestamp in the dataset differs by 1 s. Every line in the SWaT dataset comprises four main field types: 1. Timestamp: Records the exact second at which the data snapshot was taken. 2. Event type: Indicates whether the system was under normal operation or experiencing an attack. In the publicly released pre-experiment training (PET) and pre-experiment testing (PTE) splits, this is encoded as a binary label distinguishing “normal” vs. “abnormal” behavior. Specifically, PET has 495,000 consecutive samples collected over seven days of normal operation. PTE has 449,919 samples collected over four days, during which 41 distinct cyber-attack scenarios were injected (e.g., unauthorized valve manipulations, pump speed overrides, sensor spoofing). Of these, 53,900 samples (11.98%) are labeled as attacks and 396,019 samples (88.02%) as benign. 3. Operation parameters: The set points and statuses of actuators controlled by the PLCs, such as Pxxx (pumps): on/off or speed settings (e.g., P101, P301); MVxxx (motorized valves): opening degree or drive commands (e.g., MV101); and device status (sensor readings). 4. Real-time measurements from the physical and chemical monitoring devices, including LITxxx (level indicator and transmitter): water levels in tanks (e.g., LIT101, LIT301); FITxxx (flow indicator and transmitter): flow rates in pipes (e.g., FIT201, FIT401); DPITxxx (differential pressure indicator and transmitter): pressure drop across units like ultrafiltration (e.g., DPIT301); AITxxx (property indicator and transmitter): water quality metrics such as ORP and conductivity (e.g., AIT202, AIT402).

#### 5.1.3. Metrics for Assessing Anomaly Detection

In anomaly detection, data is often imbalanced, with normal samples vastly outnumbering abnormal ones, rendering simple classification accuracy inadequate. Evaluation criteria typically rely on four key metrics: True Positive (TP), False Positive (FP), False Negative (FN), and True Negative (TN). Common metrics based on these definitions include recall, precision, F1 score, and AUC.

Recall rate: Indicates the proportion of actual anomalies correctly identified, as shown in Equation (17):

(17) $$Recall=\frac{TP}{TP+FN}.$$

Precision: Indicates the proportion of detected anomalies that are true anomalies, as shown in Equation (18):

(18) $$Precision=\frac{TP}{TP+FP}.$$

F1 score: Combines recall and precision to indicate overall model performance, as shown in Equation (19):

(19) $$F1=\frac{2*(recall*precision)}{precision+recall}.$$

AUC: Represents the probability that the model ranks positive examples before negative ones, calculated as the area under the ROC curve with False Positive Rate (FPR) on the *x*-axis and True Positive Rate (TPR) on the *y*-axis.

#### 5.1.4. Baseline Algorithms

To compare with existing results, the baseline algorithms selected are TranAD, LSTM-VAE, and OmniAnomaly, and code reproduction is performed.

TranAD [25] is an anomaly detection model using a deep transformer network. It employs an attention-based sequence encoder, trained with MAML, and uses adaptive and adversarial training to enhance robustness and generalization. The algorithm is one of the few models designed to reduce computation time. Its architecture ensures quick training and detection while handling large input sequences stably and efficiently.

LSTM-VAE [34] is a multimodal anomaly detection algorithm combining LSTM and VAE. It first learns the temporal relationship of input sequences with LSTM and generates a hidden vector representation, and then learns the data distribution with VAE, and the input data is reconstructed. Finally, anomalies are detected by computing the reconstruction error and reconstruction probability.

OmniAnomaly [28] is a deep learning anomaly detection algorithm using a stochastic recurrent neural network. It provides a novel deep neural network structure to detect anomalies in multiple data sources. It combines GRUs and VAEs to detect anomalies in multivariate time series, and is the first multivariate time series anomaly detection algorithm capable of handling explicit temporal correlations between random variables. OmniAnomaly features robust performance and automatic anomaly threshold selection.

### 5.2. Experimental Analysis

#### 5.2.1. Experimental Results

**Figure 7:**
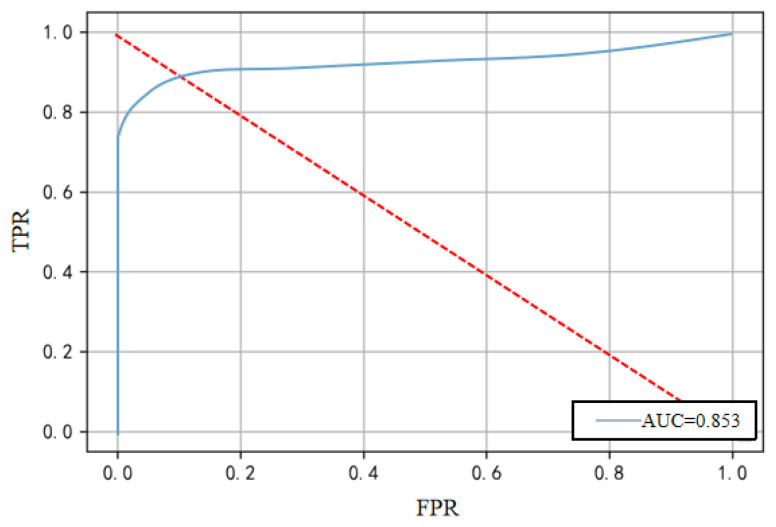
Experiment score chart.

We assess the effectiveness of our mechanism training using the experimental results in Figure 7. The AUC curve from a specific experiment yields an AUC value of 0.85, indicating excellent detection performance. This success is attributed to an enhanced encoder for improved feature extraction and an adversarial decoder that amplifies reconstruction errors.

**Figure 8:**
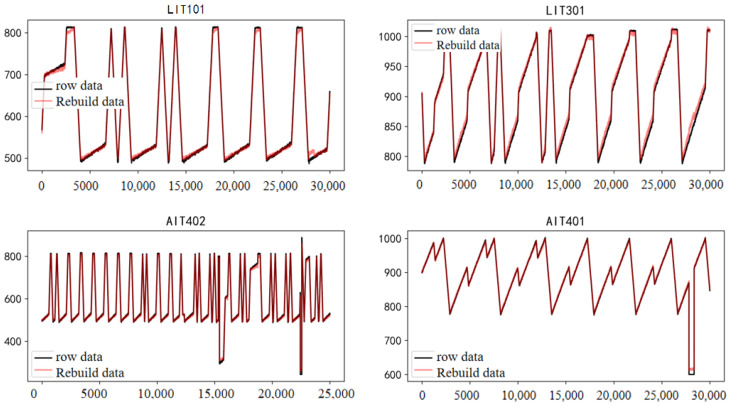
Four sensor reconstruction renderings.

To assess overall reconstruction performance, data from four sensors (LIT101, LIT301, AIT402, and AIT401) were analyzed. Figure 8 visually compares the original data (black) with reconstructed data (red). The reconstruction mechanism effectively captures complex, multi-frequency changes in AIT402 and AIT401 sensors, indicating its effectiveness.

#### 5.2.2. Comparative Experiments

To validate the improvement in the training speed of our mechanism, we conducted a comparative experiment on the detection and training times between the baseline algorithms. Detection times for the entire test set and average training times per epoch are shown in Table 2. As shown in Table 2, our mechanism exhibits some improvement in detection time. However, its impact is limited due to the already fast detection speed (2–3 s for the entire test set). Table 2 shows that our mechanism achieves a training speed 2.2 times faster per epoch than the lightweight TranAD model. This speed advantage is due to our mechanism’s simple network structure—a stack of autoencoders without additional complexities like TranAD and OmniAnomaly, reducing training time and resource usage. OmniAnomaly has the longest training time due to the many parameters required to store long-term contextual information. The LSTM-VAE model is complex because it integrates multiple fusion models. Although TranAD is specifically designed as a lightweight model to improve training speed, the additional network structures it introduces slow down training compared to our mechanism.

**Figure 9:**
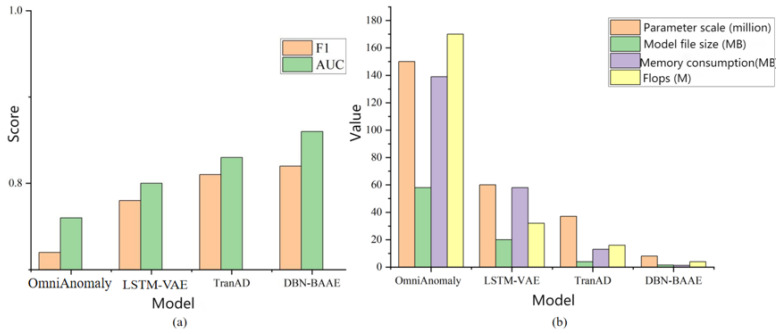
Performance comparison chart. (**a**) Averaged anomaly detection performance. (**b**) Model efficiency comparison in terms of parameters, file size, memory usage, and FLOPS.

To further evaluate DBN-BAAE’s performance, we compared it with baseline unsupervised algorithms for multivariate time series anomaly detection. Each model was tested three times for stability and the results were averaged. Comparative results are shown in Figure 9a and Table 3. Overall, our proposed mechanism outperforms similar algorithms. Specifically, our mechanism achieves better AUC, recall, precision and F1 scores than the lightweight TranAD model, attributed to enhanced encoder training for improved feature representation. OmniAnomaly performs poorly due to the absence of adversarial training, limiting its ability to detect anomalies close to normal data. LSTM-VAE shows the weakest performance, probably due to inadequate modeling of global data patterns. To validate our mechanism’s reduced resource consumption, we recorded parameters, model file size, memory usage, and floating-point operations per second (FLOPS). Our model consumes the least system resources, as shown in Figure 9b.

#### 5.2.3. Ablation Experiments

**Figure 10:**
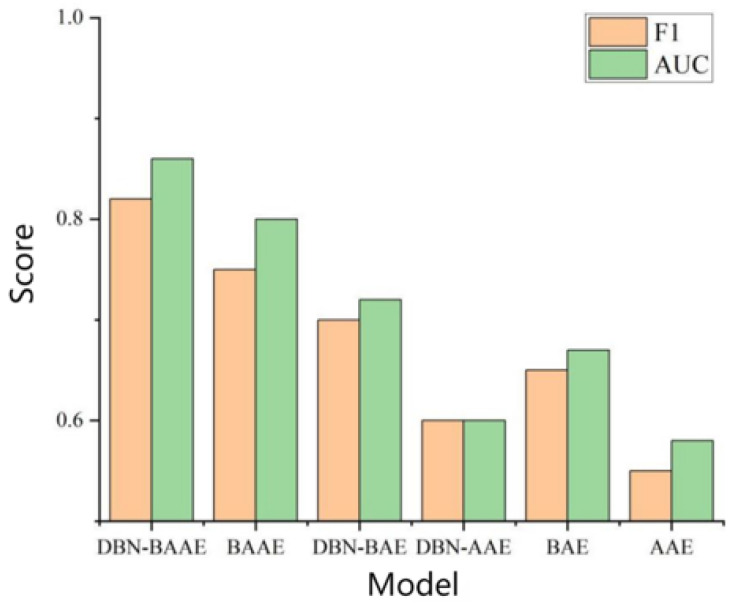
Comparison of ablation experiments.

This section proposes a combined mechanism integrating DBN pre-training to enhance the autoencoder and counter the decoder. We assessed each component’s effectiveness through experiments using control variables, with results analyzed in Figure 10. Ablating DBN from DBN-BAAE decreased both AUC and F1, highlighting the importance of a good initialization value. Ablating the adversarial decoder from the DBN-BAAE failed to detect anomalies close to normal. Ablating the enhanced encoder from the DBN-BAAE showed poor performance due to the lack of integrated learning methods to enhance reconstruction ability and reduce reconstruction error. In conclusion, the DBN-BAAE fusion mechanism exhibits superior performance, surpassing all ablation models in AUC and F1 scores.

## 6. Conclusions

This work proposes an enhanced lightweight anomaly detection mechanism based on boosting adversarial learning, termed the deep belief network-based boosting adversarial autoencoder (DBN-BAAE), which effectively addresses issues related to computational overhead, training stability, training speed, detection accuracy, and adaptive ability in existing models. Specifically, the enhanced lightweight mechanism reduces computational overhead. The improved DBN model effectively addresses the instability of autoencoder training. The proposed model enhances the encoder through ensemble learning and amplifies the decoder’s reconstruction error through adversarial techniques, significantly improving anomaly detection accuracy while maintaining fast training speed. Additionally, a dynamic threshold method is introduced to adapt to the dynamic characteristics of factory data, reducing the model retraining costs. These features make them ideally suitable for small and medium-sized factories with constrained resources, offering significant advantages in terms of overhead, stability, accuracy, and adaptability. Experimental results demonstrate that the model achieves an F1 score of 0.82 and boosts training speed by 2.2 times compared to the best-performing baseline algorithm.

Although the proposed DBN-BAAE mechanism shows promising results, there is potential for further optimization, for instance, employing data dimensionality reduction prior to algorithm training to streamline the network structure and reduce training time. Moreover, in the future, this mechanism should be applied to more datasets as well as real-time environments. Additionally, the dynamic threshold method should be refined to enhance pattern change detection accuracy, and explore the incorporation of online learning frameworks to better adapt to changing factory characteristics.

## Figures and Tables

**Table 1:** Limitations of different anomaly detection methods and relevant references.

Limitation	References
Low detection accuracy	Li et al. [24], Tuli et al. [25], Lee et al. [26]
Training instability	Su et al. [28], Park et al. [34]
High computational overhead	Su et al. [28], Li et al. [31], Yu et al. [32], Zhao et al. [33]
High memory consumption for large networks	Su et al. [28], Yu et al. [32]
Reliance on fixed thresholds, poor dynamic adaptation	Park et al. [34], Munir et al. [35], Pietroń et al. [38]
Frequent retraining needed to handle new data patterns	Munir et al. [35], Pietroń et al. [38]

**Table 2:** Detection and training time comparison.

Algorithm	Test Duration (seconds)	Training Duration per Epoch (seconds)
OmniAnomaly [28]	3.12	563
LSTM-VAE [34]	2.7	105
TranAD [25]	2.52	22
**DBN-BAAE (proposed)**	1.84	10

**Table 3:** Recall and precision comparison.

Algorithm	Recall	Precision
OmniAnomaly [28]	0.65	0.60
LSTM-VAE [34]	0.74	0.70
TranAD [25]	0.80	0.78
**DBN-BAAE (proposed)**	0.85	0.80

## Data Availability

The data sources are contained within the article.

## References

[1] Aazam M., Zeadally S., Harras K.A. (2018). Deploying fog computing in industrial internet of things and industry 4.0. IEEE Trans. Ind. Inform..

[2] Wang J., Wu H., Chen Y. (2020). Made in China 2025 and manufacturing strategy decisions with reverse QFD. Int. J. Prod. Econ..

[3] Zhu H., Liu M., Fang C., Deng R., Cheng P. (2023). Detection-Performance Tradeoff for Watermarking in Industrial Control Systems. IEEE Trans. Inf. Forensics Secur..

[4] Carpanzano E., Knüttel D. (2022). Advances in artificial intelligence methods applications in industrial control systems: Towards cognitive self-optimizing manufacturing systems. Appl. Sci..

[5] Bhamare D., Zolanvari M., Erbad A., Jain R., Khan K., Meskin N. (2020). Cybersecurity for industrial control systems: A survey. Comput. Secur..

[6] McLaughlin S., Konstantinou C., Wang X., Davi L., Sadeghi A.R., Maniatakos M., Karri R. (2016). The Cybersecurity Landscape in Industrial Control Systems. Proc. IEEE.

[7] Erfani S.M., Rajasegarar S., Karunasekera S., Leckie C. (2016). High-dimensional and large-scale anomaly detection using a linear one-class SVM with deep learning. Pattern Recognit..

[8] Zhang J., Wu D., Boulet B. Time Series Anomaly Detection for Smart Grids: A Survey. Proceedings of the 2021 IEEE Electrical Power and Energy Conference (EPEC).

[9] Hansen E.B., Bøgh S. (2021). Artificial intelligence and internet of things in small and medium-sized enterprises: A survey. J. Manuf. Syst..

[10] Obi J., Ibidunni A.S., Tolulope A., Olokundun M.A., Amaihian A.B., Borishade T.T., Fred P. (2018). Contribution of small and medium enterprises to economic development: Evidence from a transiting economy. Data Brief.

[11] Gherghina Ș.C., Botezatu M.A., Hosszu A., Simionescu L.N. (2020). Small and Medium-Sized Enterprises (SMEs): The Engine of Economic Growth through Investments and Innovation. Sustainability.

[12] Ndiaye N., Abdul Razak L., Nagayev R., Ng A. (2018). Demystifying small and medium enterprises’ (SMEs) performance in emerging and developing economies. Borsa Istanb. Rev..

[13] Gao S., Zhang Y., Jia K., Lu J., Zhang Y. (2015). Single sample face recognition via learning deep supervised autoencoders. IEEE Trans. Inf. Forensics Secur..

[14] Goodfellow I., Pouget-Abadie J., Mirza M., Xu B., Warde-Farley D., Ozair S., Courville A., Bengio Y. In Proceedings of the Advances in Neural Information Processing Systems 27 (NIPS 2014), Montreal, QC, Canada, 8–13 December 2014.

[15] Berahmand K., Daneshfar F., Salehi E.S., Li Y., Xu Y. (2024). Autoencoders and their applications in machine learning: A survey. Artif. Intell. Rev..

[16] Borji A. (2019). Pros and cons of GAN evaluation measures. Comput. Vis. Image Underst..

[17] Wu Y., Dai H.N., Tang H. (2022). Graph Neural Networks for Anomaly Detection in Industrial Internet of Things. IEEE Internet Things J..

[18] Blázquez-García A., Conde A., Mori U., Lozano J.A. (2021). A review on outlier/anomaly detection in time series data. ACM Comput. Surv. (CSUR).

[19] Shafin S.S., Karmakar G., Mareels I., Balasubramanian V., Kolluri R.R. (2024). Sensor self-declaration of numeric data reliability in internet of things. IEEE Trans. Reliab..

[20] Hinton G.E. (2009). Deep belief networks. Scholarpedia.

[21] Schapire R. (2013). Explaining adaboost. Empirical Inference.

[22] Yousef W.A., Traoré I., Briguglio W. (2021). UN-AVOIDS: Unsupervised and nonparametric approach for visualizing outliers and invariant detection scoring. IEEE Trans. Inf. Forensics Secur..

[23] Audibert J., Michiardi P., Guyard F., Marti S., Zuluaga M.A. Usad: Unsupervised anomaly detection on multivariate time series. Proceedings of the 26th ACM SIGKDD International Conference on Knowledge Discovery & Data Mining.

[24] Li J., Pedrycz W., Jamal I. (2017). Multivariate time series anomaly detection: A framework of Hidden Markov Models. Appl. Soft Comput..

[25] Tuli S., Casale G., Jennings N.R. (2022). TranAD: Deep Transformer Networks for Anomaly Detection in Multivariate Time Series Data. arXiv.

[26] Lee M.C., Lin J.C., Gran E.G. ReRe: A Lightweight Real-Time Ready-to-Go Anomaly Detection Approach for Time Series. Proceedings of the 2020 IEEE 44th Annual Computers, Software, and Applications Conference (COMPSAC).

[27] Greff K., Srivastava R.K., Koutník J., Steunebrink B.R., Schmidhuber J. (2017). LSTM: A Search Space Odyssey. IEEE Trans. Neural Netw. Learn. Syst..

[28] Su Y., Zhao Y., Niu C., Liu R., Sun W., Pei D. Robust anomaly detection for multivariate time series through stochastic recurrent neural network. Proceedings of the 25th ACM SIGKDD International Conference on Knowledge Discovery & Data Mining.

[29] Chung J., Gulcehre C., Cho K., Bengio Y. (2014). Empirical evaluation of gated recurrent neural networks on sequence modeling. arXiv.

[30] Pu Y., Gan Z., Henao R., Yuan X., Li C., Stevens A., Carin L. Variational autoencoder for deep learning of images, labels and captions. Proceedings of the Advances in Neural Information Processing Systems 29 (NIPS 2016).

[31] Li Y., Peng X., Zhang J., Li Z., Wen M. (2021). DCT-GAN: Dilated convolutional transformer-based GAN for time series anomaly detection. IEEE Trans. Knowl. Data Eng..

[32] Yu B., Yu Y., Xu J., Xiang G., Yang Z. (2023). MAG: A novel approach for effective anomaly detection in spacecraft telemetry data. IEEE Trans. Ind. Inform..

[33] Zhao H., Wang Y., Duan J., Huang C., Cao D., Tong Y., Xu B., Bai J., Tong J., Zhang Q. Multivariate Time-Series Anomaly Detection via Graph Attention Network. Proceedings of the 2020 IEEE International Conference on Data Mining (ICDM).

[34] Park D., Hoshi Y., Kemp C.C. (2018). A Multimodal Anomaly Detector for Robot-Assisted Feeding Using an LSTM-Based Variational Autoencoder. IEEE Robot. Autom. Lett..

[35] Munir M., Siddiqui S.A., Dengel A., Ahmed S. (2018). DeepAnT: A deep learning approach for unsupervised anomaly detection in time series. IEEE Access.

[36] Li Z., Liu F., Yang W., Peng S., Zhou J. (2021). A survey of convolutional neural networks: Analysis, applications, and prospects. IEEE Trans. Neural Netw. Learn. Syst..

[37] Park M.H., Chakraborty S., Vuong Q.D., Noh D.H., Lee J.W., Lee J.U., Choi J.H., Lee W.J. (2022). Anomaly detection based on time series data of hydraulic accumulator. Sensors.

[38] Pietroń M., Zurek D., Faber K. (2021). Fast and scalable neuroevolution deep learning architecture search for multivariate anomaly detection. arXiv.

[39] Castillo E. (2012). Extreme Value Theory in Engineering.

[40] Mundani R.P., Frisch J., Varduhn V., Rank E. (2015). A sliding window technique for interactive high-performance computing scenarios. Adv. Eng. Softw..

[41] Salakhutdinov R., Mnih A., Hinton G. Restricted Boltzmann machines for collaborative filtering. Proceedings of the 24th International Conference on Machine Learning.

[42] Bengio Y., Lamblin P., Popovici D., Larochelle H. Greedy layer-wise training of deep networks. Proceedings of the Advances in Neural Information Processing Systems 19 (NIPS 2006).

[43] Hinton G.E. (2002). Training Products of Experts by Minimizing Contrastive Divergence. Neural Comput..

[44] Carlo C.M. (2004). Markov chain monte carlo and gibbs sampling. Lect. Notes EEB.

[45] Abdi H., Williams L.J. (2010). Principal component analysis. Wiley Interdiscip. Rev. Comput. Stat..

[46] Dongare A., Kharde R., Kachare A.D. (2012). Introduction to artificial neural network. Int. J. Eng. Innov. Technol. (IJEIT).

[47] Dong X., Yu Z., Cao W., Shi Y., Ma Q. (2020). A survey on ensemble learning. Front. Comput. Sci..

[48] Chapelle O., Weston J., Bottou L., Vapnik V., Leen T., Dietterich T., Tresp V. (2000). Vicinal Risk Minimization. Advances in Neural Information Processing Systems.

[49] Bengio Y., Courville A., Vincent P. (2013). Representation learning: A review and new perspectives. IEEE Trans. Pattern Anal. Mach. Intell..

[50] Mathur A.P., Tippenhauer N.O. SWaT: A water treatment testbed for research and training on ICS security. Proceedings of the 2016 International Workshop on Cyber-physical Systems for Smart Water Networks (CySWater).

